# Retrocochleäre Diagnostik im Rahmen der Hörsturzuntersuchung und erfolgreiche Therapie

**DOI:** 10.1007/s00106-023-01351-5

**Published:** 2023-08-24

**Authors:** Matthias Hey, Jan Dambon, Michael Synowitz, Petra Ambrosch

**Affiliations:** 1 Klinik für Hals-Nasen-Ohren-Heilkunde, Kopf- und Halschirurgie, UKSH Campus Kiel, Arnold-Heller-Straße 14, 24105 Kiel, Deutschland; 2 Klinik für Neurochirurgie, UKSH Campus Kiel, Kiel, Deutschland

**Keywords:** Hörminderung, Hypakusis, Audiologie, Meningeom, Audiometrie, Hearing loss, Hypacusis, Audiology, Meningioma, Audiometry

## Abstract

Eine 41-jährige Patientin stellte sich wegen einer vor drei Monaten akut aufgetretenen und seitdem persistierenden einseitigen Hörminderung vor. Bei dem Verdacht auf einen Hörsturz auf dem rechten Ohr wurde vorab eine systemische Therapie mit oralen Glukokortikoiden in absteigender Dosierung durchgeführt, die zu keiner Verbesserung führte. Im Rahmen der audiologischen Diagnostik wurde der Verdacht einer retrocochleären Hörstörung gestellt. Durch bildgebende Diagnostik wurde ein Meningeom diagnostiziert. Die nachfolgende operative Entfernung erzielte eine deutliche Hörverbesserung.

## Abstract (en)

A 41-year-old female patient presented due to acute onset of unilateral hearing loss 3 months previously and persistent since then. Systemic therapy with oral glucocorticoids in decreasing doses had been performed beforehand, but did not lead to any improvement. In the course of audiological diagnostics, based on subjective and objective methods, a retrocochlear hearing disorder was suspected. A meningioma was diagnosed by diagnostic imaging. Subsequent surgical removal achieved a significant hearing improvement.

## Anamnese und Befund

Eine 41-jährige Patientin stellte sich wegen einer vor drei Monaten akut aufgetretenen und seitdem persistierenden rechtsseitigen Hörminderung vor. Bei dem Verdacht auf einen Hörsturz auf dem rechten Ohr sei bereits eine systemische Therapie mit oralen Glukokortikoiden in absteigender Dosierung durchgeführt worden. Hierunter habe sich zunächst keine weitere Dynamik der Hörminderung gezeigt. Darüber hinaus berichtete die Patientin über ein rechtsseitiges Ohrgeräusch (stationäres Rauschen). Weitere ohrspezifische Symptome wie Otorrhö, Otalgie oder Vertigo wurden verneint. An Nebendiagnosen ist neben einer Hypothyreose eine Psoriasis bekannt.

**Figure 1:**
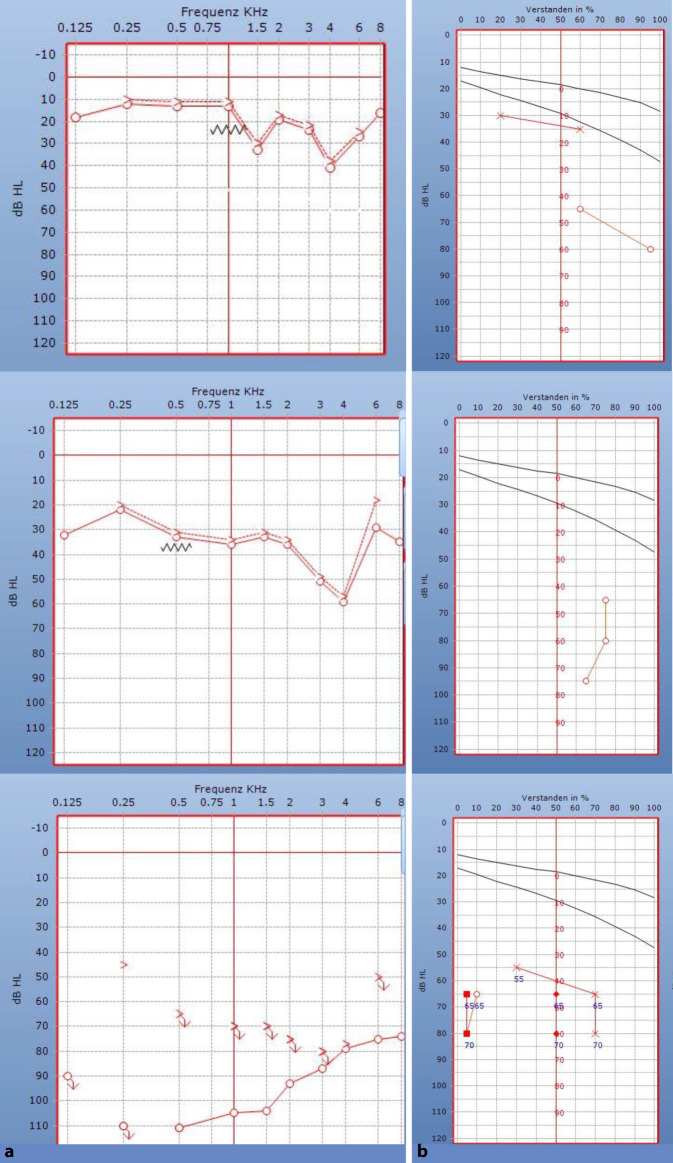

**Figure 2:**
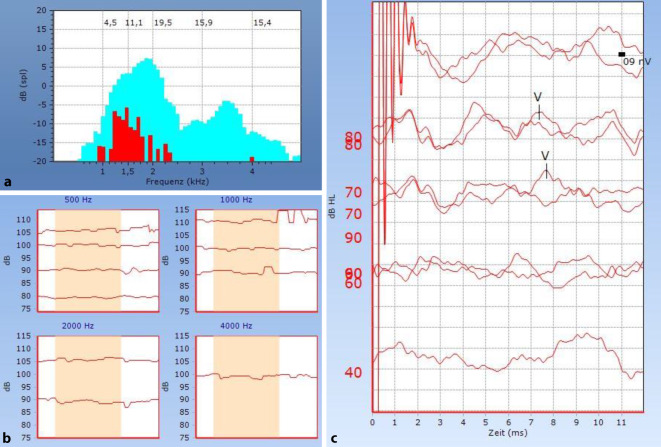

Die Trommelfelle waren beidseits reizlos und die Mittelohren belüftet. In der Reintonaudiometrie zeigte sich bei Erstvorstellung rechtsseitig eine geringgradige hochtonbetonte Innenohrschwerhörigkeit und linksseitig eine Normakusis. Sprachaudiometrisch lag ein Einsilberverstehen bei 65 dB von 60 % und bei 80 dB von 95 % vor (Abb. 1, oben). In der Tympanometrie zeigte sich beidseits eine spitzgipflige Trommelfell-Compliance (Typ A nach Jerger), wobei die ipsilateralen Stapediusreflexe rechtsseitig nicht auslösbar waren (Abb. 2b). Die transitorisch evozierten otoakustischen Emissionen waren im Frequenzbereich 1–4 kHz nachweisbar (Reproduzierbarkeit = 95 %; Stabilität = 99 %; Abb. 2a). Aufgrund der Diskrepanz zwischen den subjektiven Verfahren Ton- und Sprachaudiometrie einerseits und den otoakustischen Emissionen und den Stapediusreflexmessungen andererseits wurde eine kurzfristige Wiedervorstellung zur audiometrischen Kontrolle vereinbart. Hierbei konnte in der Sprachaudiometrie weiterhin ein persistierend reduziertes Einsilberverstehen sowie bei nun pantonal leicht- bis mittelgradiger Schwerhörigkeit in der Reintonaudiometrie eine Progredienz des Hörverlusts festgestellt werden (Abb. 1 mittig). Die im Rahmen der Kontrolluntersuchung ergänzte Hirnstammaudiometrie („auditory brainstem response“, ABR) ergab eine Nachweisschwelle der Welle V rechts bei 70 dB. Die Absolutlatenzen der Welle V waren mit einer Seitendifferenz von im Mittel 2,1 ms im Vergleich zur Gegenseite pathologisch verlängert. Die Interpeaklatenzen der Welle I–V waren nicht auswertbar (Abb. 2c).

Unter der Frenzel-Brille zeigte sich weder ein Spontan- noch ein Provokationsnystagmus. Die weitere vestibuläre Funktionsdiagnostik zeigte im Video-Kopfimpulstest pathologische Covert-Sakkaden im rechten lateralen Bogengang sowie einen verminderten Gain mit relativer Asymmetrie von 21 %. Die übrigen Bogengänge stellen sich unauffällig dar. In der kalorischen Prüfung zeigte sich eine Mindererregbarkeit des rechten lateralen Bogengangs mit einer Seitendifferenz von 40 %. Die Funktion des N. facialis war seitengleich uneingeschränkt.

Zusammenfassend zeigten sich folgende Auffälligkeiten in den audiologischen und neurootologischen Befunden:

- Diskrepanz zwischen Ton- und Sprachaudiometrie,
- TEOAE nachweisbar bei einer mittelgradigen sensorineuralen Schwerhörigkeit,
- ABR-Schwelle deutlich höher, als aus dem Tonaudiogramm zu erwarten,
- pathologische Seitendifferenz der Absolutlatenzen der Welle V von > 0,5 ms,
- keine Stapediusreflexe nachweisbar bei einer mittelgradigen sensorineuralen Schwerhörigkeit,
- verminderte Erregbarkeit im Kopfimpulstest sowie in der Kalorik ohne subjektives Schwindelgefühl.

Es wurde die Verdachtsdiagnose einer retrocochleären Hörstörung gestellt und eine MRT des Gehirns veranlasst.

## Verlauf und Therapie

**Figure 3:**
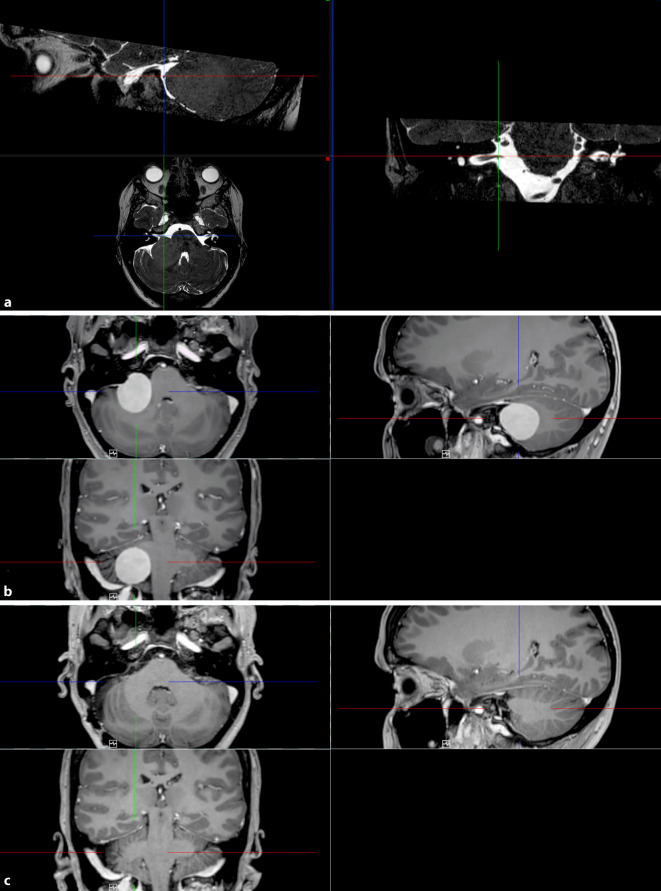

MR-tomographisch kam als erklärende Pathologie eine ca. 3 × 3 × 3 cm durchmessende extraaxiale Raumforderung im Kleinhirnbrückenwinkel rechts mit Verlagerung von Kleinhirn und Hirnstamm sowie konsekutivem diskretem zerebellärem Ödem zur Darstellung (Abb. 3). Radiologisch wurde der Verdacht auf ein Meningeom der hinteren Schädelgrube geäußert. Das verdrängende Wachstum der Raumforderung wirkte sich auf das Hörvermögen als retrocochleäre Hörstörung aus. Eine operative Entfernung des Kleinhirnbrückenwinkeltumors durch die Neurochirurgie wurde angebahnt.

Bei präoperativer Vorstellung (zwei Monate nach Erstvorstellung) zur audiometrischen Verlaufskontrolle zeigte sich in der Reintonaudiometrie eine hochgradige, in den tiefen Frequenzen an Taubheit grenzende Schwerhörigkeit (Abb. 1, unten). Sprachaudiometrisch kam es zwischenzeitlich weiterhin zu einer Verschlechterung des Einsilberverstehens auf nunmehr 15 % bei 65 dB und 10 % bei 80 dB (Abb. 1, unten). Die TEOAE waren weiterhin rechtsseitig nachweisbar (Reproduzierbarkeit = 97 %; Stabilität = 100 %).

Es erfolgte die mikrochirurgische Exstirpation der Raumforderung im Kleinhirnbrückenwinkel über einen mittleren retrosigmoidalen Zugang rechts. Die neuropathologische Untersuchung ergab ein meningotheliomatöses Meningeom (WHO Grad I°) mit erhöhter Proliferation.

Es wurde drei Monate postoperativ eine Verlaufskontrolle mittels kranieller MRT-Untersuchung nativ und nach Kontrastmittelgabe durchgeführt. Hierbei zeigte sich kein Hinweis auf ein Tumorresiduum oder -rezidiv. Die bildgebende Verlaufsuntersuchung ein Jahr nach der Operation ergab einen tumorfreien Befund.

**Figure 4:**
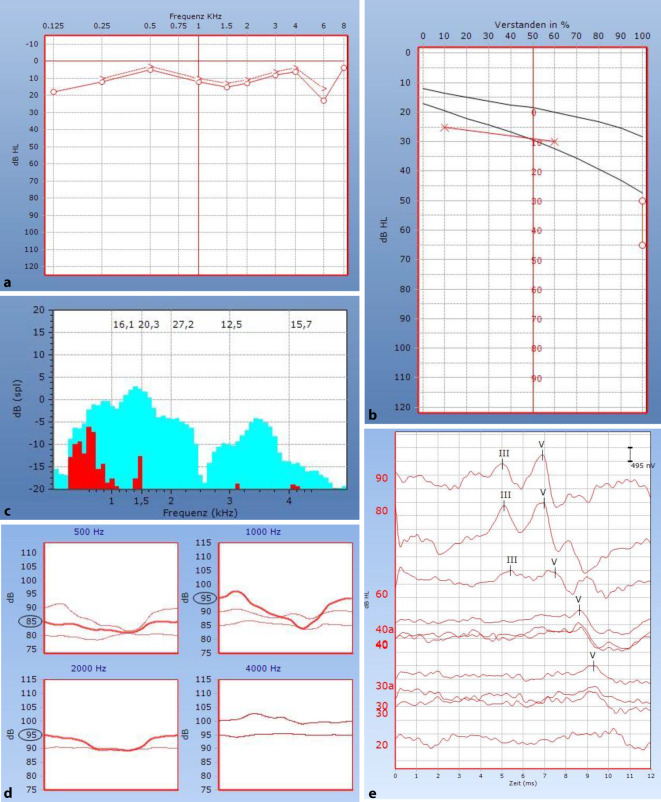

Im Rahmen der postoperativen audiologischen Diagnostik konnte eine wieder normalisierte Funktion des Hörens und Verstehens auf dem rechten Ohr nachgewiesen werden (Abb. 4). Im Tonaudiogramm ist kein substanzieller Hörverlust in den Prüffrequenzen 125–8000 Hz nachzuweisen (Hörverlust < 25 dB). Der Hörverlust für Zahlen ist damit in Deckung (HVZ = 10 dB), und es wird bei 50 und 65 dB das maximale Wortverstehen von 100 % erreicht. TEOAE sind weiterhin nachweisbar. Stapediusreflexe in den Frequenzen 500–2000 Hz sind nun erstmals zu erkennen. Die ABR-Schwelle ist rechtsseitig bei ≤ 20 dB zu finden. Lediglich die Absolutlatenzen der Welle V sind im Mittel um 1,4 ms im Vergleich zur normalhörigen Gegenseite verlängert. Im Vergleich zum präoperativen Befund hat sich diese Seitendifferenz um 0,7 ms verbessert.

Die Zusammenschau dieser Befunde zeigt postoperativ keinen Anhalt mehr für eine periphere vestibulocochleäre Störung rechts. Durch die Entfernung des Tumors ist offensichtlich eine Dekompression des N. vestibulocochlearis erfolgt, was zur fast vollständigen Funktionswiederkehr geführt hat.

## Diskussion

Meningeome stellen die häufigste Entität intrakranieller Tumoren bei Erwachsenen [1, 2] und nach den Vestibularisschwannomen (~85 %) die zweithäufigste im Bereich des Kleinhirnbrückenwinkels dar (5–15 %) [3–5]. Ein gehäuftes Auftreten wurde bei Frauen in der 5. Lebensdekade beschrieben [6, 7]. In der histopathologischen Aufarbeitung zeigen sich 80–90 % dieser Tumoren – wie im dargestellten Fall – benigne (WHO Grad I°) und vom meningotheliomatösen Subtyp [8, 9]. Die Tumorgröße, Ausdehnung in den Meatus acusticus internus sowie ein perifokal auftretendes Hirnödem wurden als Risikofaktoren für Komplikationen bei einer chirurgischen Resektion identifiziert [10].

Obwohl Meningeome des Kleinhirnbrückenwinkels benigne Tumoren sind, können sie degenerative Veränderungen des N. facialis und des N. vestibulocochlearis bewirken [1]. Erste klinische Manifestation ist häufig eine Hörminderung mit Tinnitus [11]. Retrocochleäre Hörstörungen – allen voran die Vestibularisschwannome – können zu einer Diskrepanz von Einsilberverstehen und Reintonaudiogramm führen [12]. In der objektiven Audiometrie können verlängerte Latenzen sowie erhöhte Schwellen in der Hirnstamm- und Reflexaudiometrie den Verdacht auf eine retrocochleäre Hörstörung erhärten.

Darüber hinaus können Auffälligkeiten in der neurootologischen Untersuchung richtungsweisend sein für eine retrocochleäre Hörstörung. Dabei kann eine Mindererregbarkeit des Vestibularorgans – wie in diesem Fall – auch ohne subjektives Schwindelgefühl auftreten und mittels Kalorik respektive Kopfimpulstest objektiviert werden. Bei Vestibularisschwannomen zeigt sich hierbei häufiger ein auffälliger Befund in der Kalorik als bei anderen Kleinhirnbrückenwinkeltumoren [13]. Eine ätiologische Differenzierung mithilfe neurootologischer Befunde ist hingegen nicht möglich [14]. Ebenso schließt eine Spontanremission der Hörminderung das Vorliegen eines Tumors im Kleinhirnbrückenwinkel nicht aus [15, 16]. Bei Verdacht auf retrocochleäre Hörstörung ist eine kranielle Bildgebung mittels MRT der Goldstandard [2, 17, 18].

In einer histopathologischen Post-mortem-Studie konnte eine substanzielle cochleäre Schädigung, wie sie für Vestibularisschwannome beschrieben ist [19], für intrameatale Meningeome nicht nachgewiesen werden [20, 21]. Während otoakustische Emissionen bei Vestibularisschwannomen pathologisch sein können, kommen diese bei Meningeomen trotz erhöhter Hörschwelle regelmäßig zur Darstellung [22]. Darüber hinaus konnte mit der audiometrischen Hörschwellenkurve keine Vorhersage zum cochleären Schaden auf zellulärer Ebene getroffen werden [23]. Korrespondierend mit diesen histopathologischen Studien wurden in den vergangenen Jahrzehnten mehrfach Fallberichte zur Remission einer Hörschädigung nach operativer Entfernung eines Kleinhirnbrückenwinkelmeningeoms publiziert [15, 22, 24–26]. Als maßgebender Faktor für die postoperativ zu erwartende Hörverbesserung wurde das präoperative Hörvermögen, gemessen mittels Reintonaudiogramm und Sprachdiskrimination, identifiziert [6, 27]. In der hierzu bislang größten publizierten retrospektiven Studie (*n* = 421) [27] konnte bei lediglich 1,8 % der präoperativ hochgradig, an Taubheit grenzend schwerhörigen Patienten eine Hörverbesserung erzielt werden. Eine komplette Reversibilität im Sinne einer wiedererlangten Normakusis konnte in keinem dieser Fälle postoperativ nachgewiesen werden.

Am vorliegenden Fall soll exemplarisch aufgezeigt werden, dass Diskrepanzen zwischen der Ton- und Sprachaudiometrie ein entscheidendes Verdachtsmoment für eine retrocochleäre Hörstörung darstellen können. Eine daraufhin frühzeitig initiierte Bildgebung ermöglichte eine rasche Diagnosestellung und zielgerichtete Therapie. Durch die operative Tumorexstirpation [2] und somit Dekompression des N. vestibulocochlearis war in diesem Fall trotz präoperativ hochgradiger Hörminderung eine Restitutio ad integrum möglich. Diese Normalisierung konnte sowohl durch subjektive (Ton- und Sprachaudiometrie) als auch durch objektive (Stapediusreflexe, Hirnstammaudiometrie) audiometrische Untersuchungsverfahren bestätigt werden.

## Fazit für die Praxis

- Diskrepanzen zwischen Ton- und Sprachaudiometrie sollten frühzeitig Anlass zur weiteren Abklärung einer retrocochleären Hörstörung geben.
- In diesen Fällen sind die otoakustischen Emissionen, akustisch evozierte Potenziale, Stapediusreflexe, Kopfimpulstest und Kalorik hilfreiche objektive diagnostische Verfahren.
- Eine MRT des Gehirns bleibt Goldstandard in der Diagnostik einer retrocochleären Hörstörung.
- Bei Meningeomen besteht die Chance auf deutliche Hörverbesserung durch operative Tumorentfernung.

## References

[1] McNeill KA (2016). Epidemiology of Brain Tumors. Neurol Clin.

[2] Goldbrunner R, Stavrinou P, Jenkinson MD (2021). EANO guideline on the diagnosis and management of meningiomas. Neuro Oncol.

[3] Alonso Seco A, Polo López R, Labatut Pesce T (2010). Meningioma of the internal auditory canal: A rare entity. Acta Otorrinolaringol Esp.

[4] Tomogane Y, Mori K, Izumoto S (2013). Usefulness of PRESTO magnetic resonance imaging for the differentiation of schwannoma and meningioma in the cerebellopontine angle. Neurol Med Chir (tokyo).

[5] Friedmann DR, Grobelny B, Golfinos JG (2015). Nonschwannoma tumors of the cerebellopontine angle. Otolaryngol Clin North Am.

[6] Roser F, Nakamura M, Dormiani M (2005). Meningiomas of the cerebellopontine angle with extension into the internal auditory canal. J Neurosurg.

[7] Raper DMS, Starke RM, Henderson F (2014). Preoperative embolization of intracranial meningiomas: efficacy, technical considerations, and complications. AJNR Am J Neuroradiol.

[8] He S, Pham MH, Pease M (2013). A review of epigenetic and gene expression alterations associated with intracranial meningiomas. Neurosurg Focus.

[9] Domingues PH, Sousa P, Otero Á (2014). Proposal for a new risk stratification classification for meningioma based on patient age, WHO tumor grade, size, localization, and karyotype. Neuro Oncol.

[10] Gao K, Ma H, Cui Y (2015). Meningiomas of the cerebellopontine angle: radiological differences in tumors with internal auditory canal involvement and their influence on surgical outcome. PLoS ONE.

[11] Gerganov V, Bussarsky V, Romansky K (2003). Cerebellopontine angle meningiomas: Clinical features and surgical treatment. J Neurosurg Sci.

[12] Kemper M, Paliege K, Zahnert T (2022). Vestibular schwannomas—baseline and progress diagnostics. Laryngorhinootologie.

[13] Hu YF, Cheng PW, Young YH (2009). Comparison of vestibular function between large cerebellopontine angle meningioma and schwannoma. Acta Otolaryngol.

[14] Su CH, Chen CM, Young YH (2013). Differentiating cerebellopontine angle meningioma from schwannoma using caloric testing and vestibular-evoked myogenic potentials. J Neurol Sci.

[15] Christiansen CB, Greisen O (1975). Reversible hearing loss in tumours of the cerebello-pontine angle. J Laryngol Otol.

[16] Berenholz LP, Eriksen C, Hirsh FA (1992). Recovery from repeated sudden hearing loss with corticosteroid use in the presence of an acoustic neuroma. Ann Otol Rhinol Laryngol.

[17] Fortnum H, O’Neill C, Taylor R (2009). The role of magnetic resonance imaging in the identification of suspected acoustic neuroma: a systematic review of clinical and cost effectiveness and natural history. Health Technol Assess.

[18] Waterval J, Kania R, Somers T (2018). EAONO Position statement on vestibular schwannoma: Imaging assessment. What are the indications for performing a screening MRI scan for a potential vestibular schwannoma?. J Int Adv Otol.

[19] Roosli C, Linthicum FH, Cureoglu S (2012). Dysfunction of the cochlea contributing to hearing loss in acoustic neuromas: an underappreciated entity. Otol Neurotol.

[20] Landegger LD, Lee JD, Linthicum FH (2017). Cochlear dysfunction is not common in human meningioma of the internal auditory canal. Otol Neurotol.

[21] Salvinelli F, Trivelli M, Greco F (1999). Acoustic neuromas and meningiomas. Histopathological aspect: A post mortem study on temporal bones. Eur Rev Med Pharmacol Sci.

[22] Kileny PR, Edwards BM, Disher MJ (1998). Hearing improvement after resection of cerebellopontine angle meningioma: case study of the preoperative role of transient evoked otoacoustic emissions. J Am Acad Audiol.

[23] Landegger LD, Psaltis D, Stankovic KM (2016). Human audiometric thresholds do not predict specific cellular damage in the inner ear. Hear Res.

[24] Maurer PK, Okawara SH (1988). Restoration of hearing after removal of cerebellopontine angle meningioma: diagnostic and therapeutic implications. Neurosurgery.

[25] Goebel JA, Vollmer DG (1993). Hearing improvement after conservative approach for large posterior fossa meningioma. Otolaryngol Head Neck Surg.

[26] Kashio A, Suzuki M (2007). Bilateral hearing loss due to a meningioma located in the left posterior fossa: a case report. Acta Otolaryngol Suppl.

[27] Nakamura M (2014). Facial and cochlear nerve function after surgery of cerebellopontine angle meningiomas. Samii’s Essentials in. Neurosurgery.

